# EP_1_ receptor antagonism mitigates early and late stage renal fibrosis

**DOI:** 10.1111/apha.13780

**Published:** 2022-01-30

**Authors:** Jean‐Claude Kresse, Henricus A. M. Mutsaers, Michael Schou Jensen, Stine Julie Tingskov, Mia Gebauer Madsen, Lene N. Nejsum, Helle Prætorius, Rikke Nørregaard

**Affiliations:** ^1^ Department of Clinical Medicine Aarhus University Aarhus Denmark; ^2^ Department of Urology Aarhus University Hospital Aarhus Denmark; ^3^ Department of Biomedicine Aarhus University Aarhus Denmark

**Keywords:** chronic kidney disease, human precision‐cut kidney slices, prostaglandin E_2_ EP_1_ receptor, renal fibrosis

## Abstract

**Aim:**

Renal fibrosis is a major driver of chronic kidney disease, yet current treatment strategies are ineffective in attenuating fibrogenesis. The cyclooxygenase/prostaglandin system plays a key role in renal injury and holds great promise as a therapeutic target. Here, we used a translational approach to evaluate the role of the PGE_2_‐EP_1_ receptor in the pathogenesis of renal fibrosis in several models of kidney injury, including human (fibrotic) kidney slices.

**Methods:**

The anti‐fibrotic efficacy of a selective EP_1_ receptor antagonist (SC‐19220) was studied in mice subjected to unilateral ureteral obstruction (UUO), healthy and fibrotic human precision‐cut kidney slices (PCKS), Madin‐Darby Canine Kidney (MDCK) cells and primary human renal fibroblasts (HRFs). Fibrosis was evaluated on gene and protein level using qPCR, western blot and immunostaining.

**Results:**

EP_1_ receptor inhibition diminished fibrosis in UUO mice, illustrated by a decreased protein expression of fibronectin (FN) and α‐smooth muscle actin (αSMA) and a reduction in collagen deposition. Moreover, treatment of healthy human PCKS with SC‐19220 reduced TGF‐β‐induced fibrosis as shown by decreased expression of collagen 1A1, FN and αSMA as well as reduced collagen deposition. Similar observations were made using fibrotic human PCKS. In addition, SC‐19220 reduced TGF‐β‐induced FN expression in MDCK cells and HRFs.

**Conclusion:**

This study highlights the EP_1_ receptor as a promising target for preventing both the onset and late stage of renal fibrosis. Moreover, we provide strong evidence that the effect of SC‐19220 may translate to clinical care since its effects were observed in UUO mice, cells and human kidney slices.

## 1 INTRODUCTION

Chronic kidney disease (CKD) is a leading cause of death and the global prevalence is currently estimated to be about 10%‐15%.^1^ Regardless of the underlying cause, CKD is characterized by fibrotic changes in the kidney and progressive loss of renal function, which can ultimately lead to end‐stage renal disease (ESRD).^2^ Persistent fibrogenesis is regarded as the most important pathologic process underlying the progression of CKD. Despite overwhelming efforts to find therapeutics that arrest the fibrotic process, current treatment strategies have proven ineffective in attenuating renal fibrogenesis. Thus, the identification of novel therapeutic targets is of the utmost importance.

Inflammatory processes mediated by the cyclooxygenase/prostaglandin (COX/PG) system play fundamental roles in the progression of renal injury.^3^, ^4^, ^5^, ^6^, ^7^, ^8^, ^9^, ^10^ Prostaglandin E_2_ (PGE_2_) is the major prostaglandin in the kidney, and it is involved in the regulation of several physiological processes such as renal haemodynamics and water and salt homeostasis.^11^, ^12^ The physiological effects of PGE_2_ are mediated via the G‐protein‐coupled receptors EP_1‐4_,^13^ and thus, the most likely target for PGE_2_ effects during renal injury. We have recently reported that activation of the EP_2_ receptor with butaprost mitigates renal fibrosis in mice subjected to unilateral ureteral obstruction (UUO), MDCK cells and human precision‐cut kidney slices.^14^ In addition, EP_4_ agonism has been shown to have anti‐fibrotic effects in UUO mice and cultured renal fibroblasts.^15^ Furthermore, it has been reported that antagonism or deletion of the EP_1_ receptor has therapeutic potential by reducing renal fibrosis in diabetic mice^16^ and hypertensive rats.^17^ In contrast, EP_1_ deletion caused severe renal impairment in glomerulonephritic mice.^18^ Thus, the role of the EP_1_ receptor in kidney diseases remains unclear. Based on previous work, we hypothesize that EP_1_ receptor antagonism will reduce renal fibrosis.

In this study, we used a translational approach to investigate the impact of SC‐19220, an EP_1_ receptor antagonist, on renal fibrosis. To this end, we used a combination of well‐known in vitro and in vivo fibrosis models as well as a novel ex vivo fibrosis model, namely human precision‐cut kidney slices (PCKS). This unique model is suitable for studying multicellular (pathological) processes, including fibrosis, directly in human kidney tissue as cellular heterogeneity and organ architecture is preserved in the slices.^19^, ^20^ Moreover, we studied the impact of SC‐19220 on the late stage of fibrogenesis using PCKS prepared from patients with established renal fibrosis.

## 2 RESULTS

### 2.1 EP_1_ receptor expression is not altered in response to UUO in mice

**FIGURE 1:**
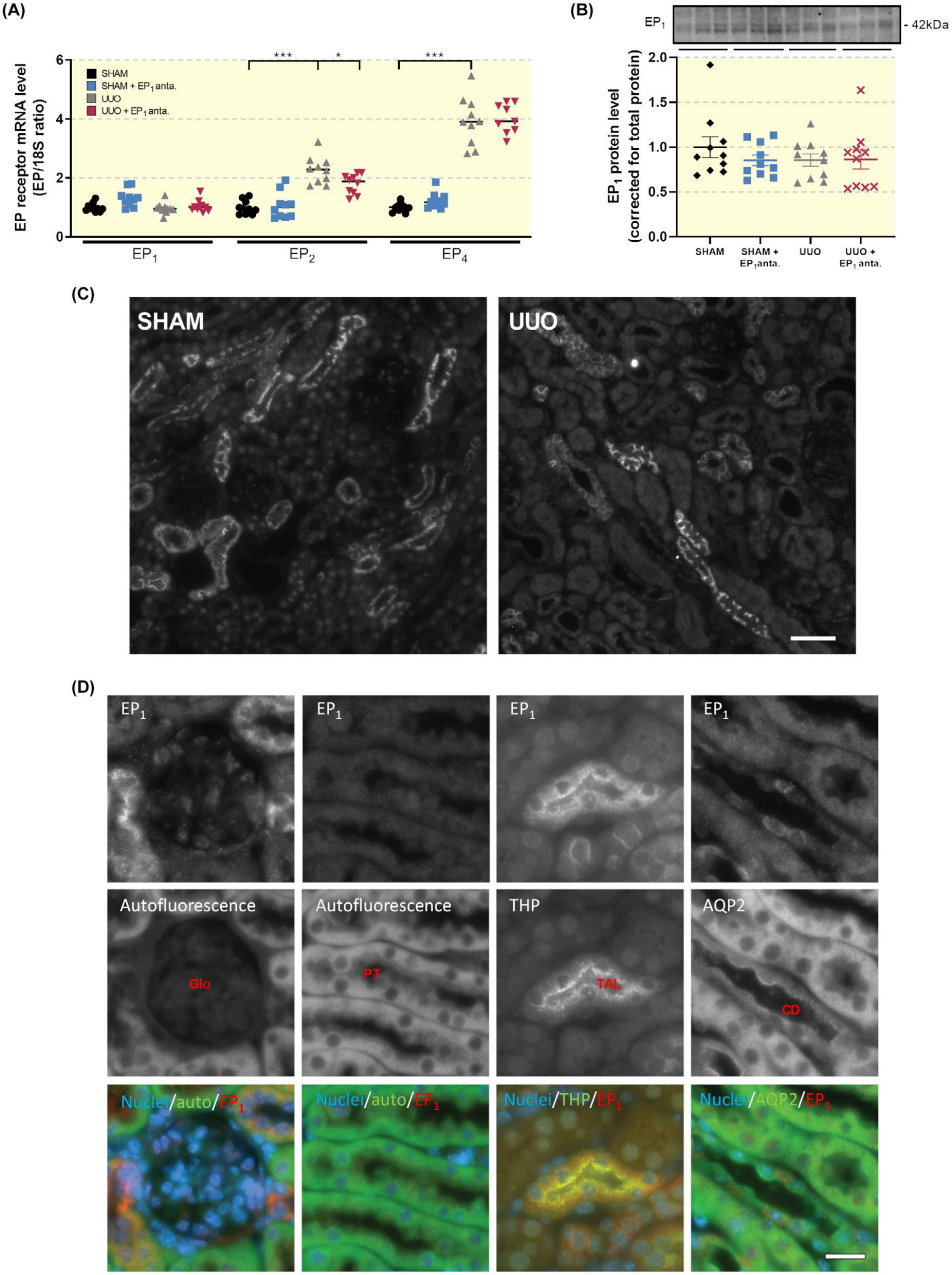
Renal EP_1_ receptor expression and localization does not change following UUO. (A) EP receptor gene expression was studied by qPCR. Relative expression was calculated using the reference gene 18S (n = 10). (B) EP_1_ protein expression was studied using western blotting. Protein levels were calculated in relation to total protein (n = 10). Data are presented as means ± SEM. (C) Representative immunofluorescent images of EP_1_ stainings of sham and UUO mice (n = 3). Scale bar is 50 µm. (D) Representative images of renal autofluorescence captured with the FITC imaging setting as well as immunofluorescent staining of EP_1_, Tamm Horsfall protein (THP) and aquaporin‐2 (AQP2). Double stainings were EP_1_ and THP (panel 2) and EP_1_ and AQP2 (panels 1, 3 and 4). Stainings are shown in inverted contrast, whereas in the merged images, cell nuclei are shown in blue, EP_1_ in red and autofluorescence as well as THP and AQP2 are shown in green. Scale bar is 20 μm

First, we set out to evaluate the potential anti‐fibrotic effect of SC‐19220 in vivo. To this end, we first investigated whether UUO affected the gene expression of the EP receptors using qPCR and protein expression of the EP_1_ receptor using WB and IHC. After 7 days of UUO, mRNA levels of both EP_2_ and EP_4_ markedly increased in UUO mice (Figure 1A), in line with the previous observations.^9^ Conversely, both mRNA and protein levels of the EP_1_ receptor were unchanged as compared to sham mice (Figure 1A,B). Immunofluorescence staining of the EP_1_ receptor showed labelling in the collecting ducts (CDs) and thick ascending limbs (TALs; Figure 1D). Additionally, the EP_1_ receptor was detected in the glomeruli (Figure 1D). No obvious differences in staining intensity of the EP_1_ receptor between sham and UUO mice were observed (Figure 1C, visual inspection).

### 2.2 EP_1_ receptor antagonism ameliorates UUO‐induced fibrosis

**FIGURE 2:**
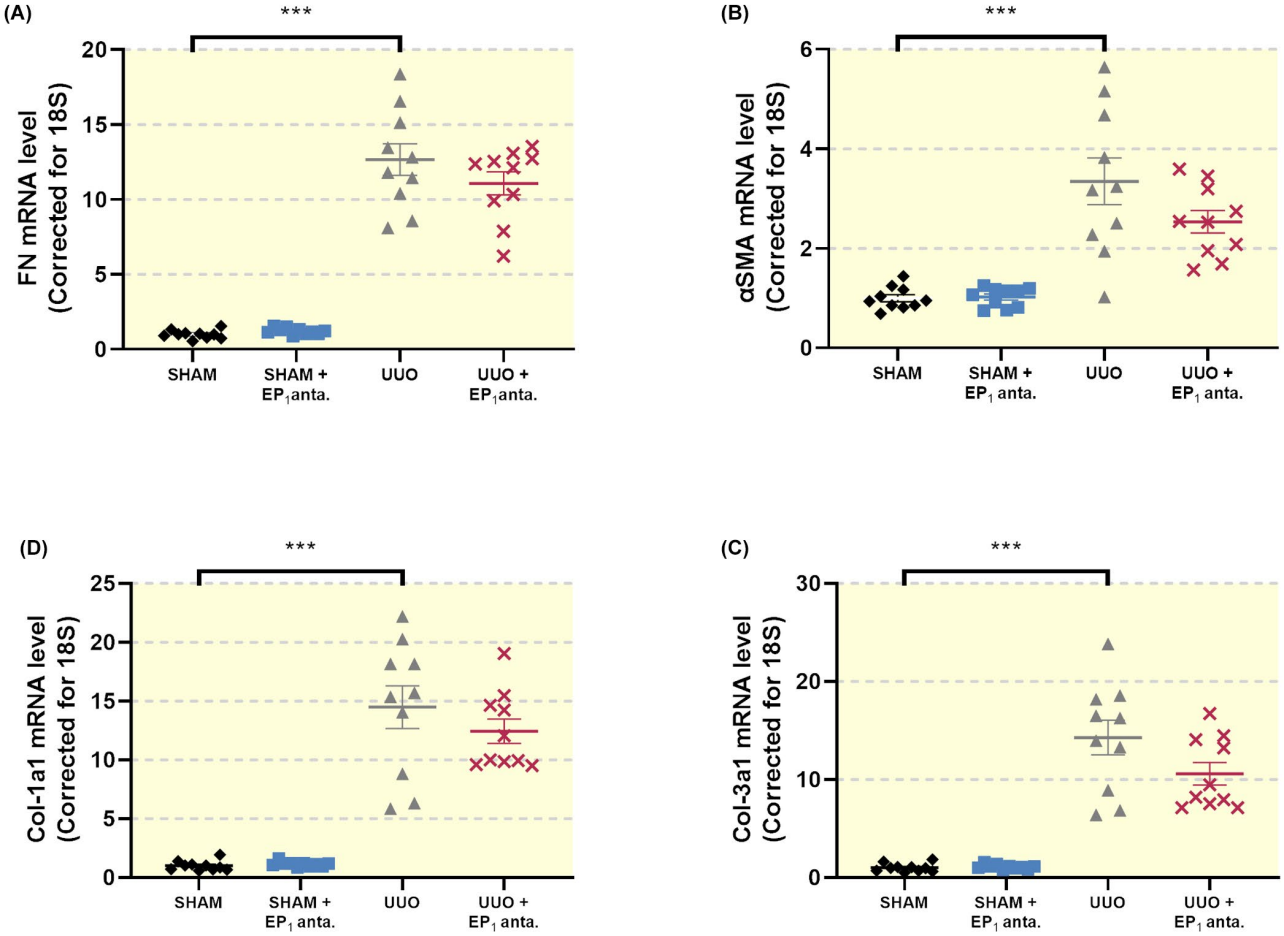
EP_1_ receptor inhibition does not alter the gene expression of fibrotic markers in UUO mice. Mice were subject to 7 days of UUO and treated with SC‐19220 (25 mg/kg) via IP injection. Afterwards, gene expression of (A) fibronectin (FN), (B) alpha‐smooth muscle actin (αSMA), (C) collagen 1a1 (Col‐1a1) and (D) collagen 3a1 (Col‐3a1) was studied by qPCR. Relative expression was calculated using the reference gene 18S (n = 10). Data are presented as means ± SEM; ***P < .001

**FIGURE 3:**
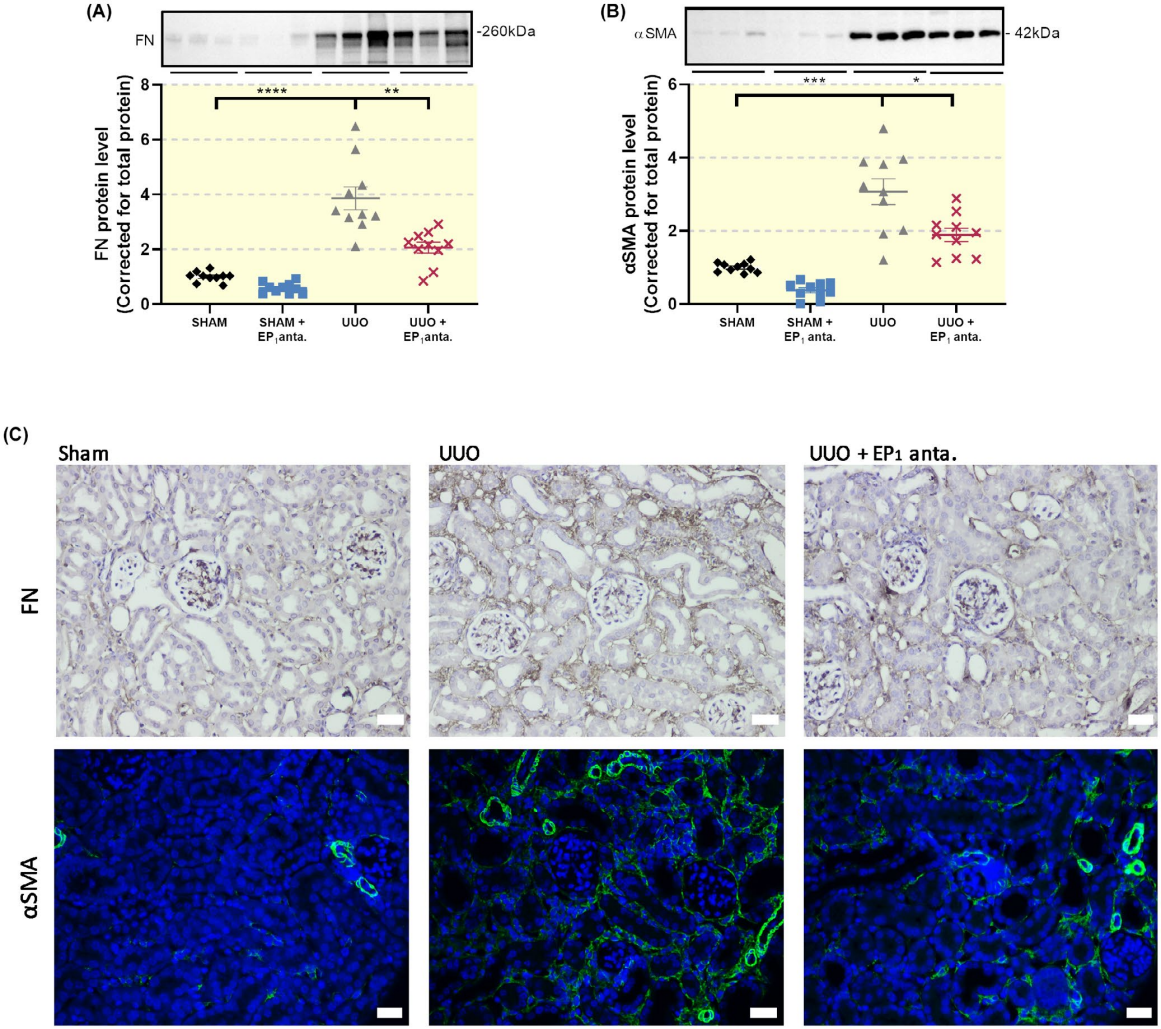
Inhibition of the EP_1_ receptor reduces UUO‐induced protein expression of fibrotic markers. Mice were subject to 7 days of UUO and treated with SC‐19220 (25 mg/kg) via IP injection, followed by Western Blot analysis of fibrotic markers (A) fibronectin (FN) and (B) alpha‐smooth muscle actin (αSMA) (n = 10). Data are presented as means ± SEM; *P < .05, **P < .01 and ***P < .001. (C) Representative immunohistochemistry images of FN (upper) and αSMA (lower) expression, 20× magnification, scale bar is 50 μm. n = 4

**FIGURE 4:**
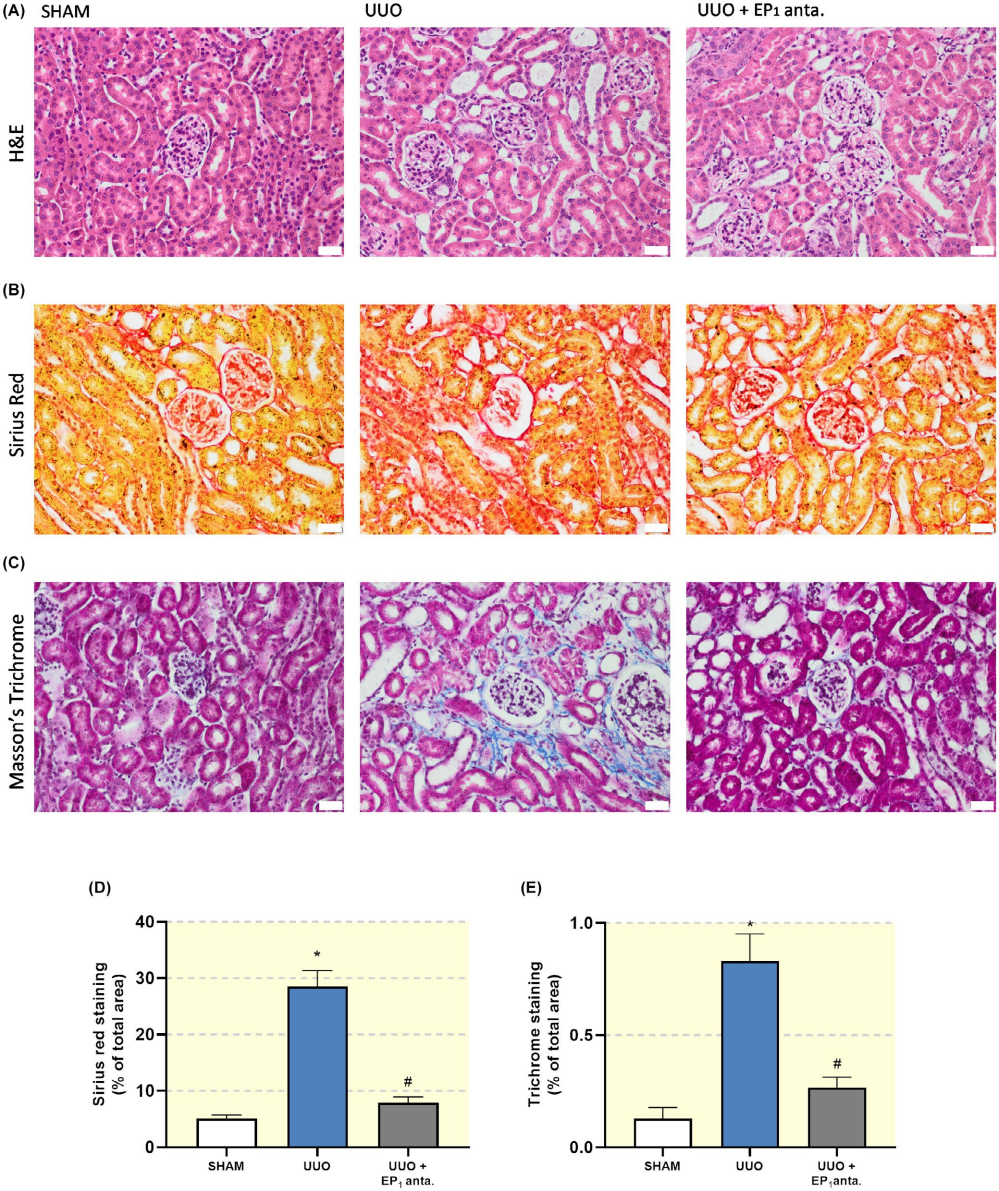
EP_1_ receptor antagonism mitigates UUO‐induced renal injury. Mice were subject to 7 days of UUO and treated with SC‐19220 (25 mg/kg) via IP injection. Afterwards, renal injury was assessed by IHC. Representative microscopy images of kidney tissue following (A) Mayers haematoxylin and eosin staining, (B) Sirius red staining, (C) Masson's trichrome staining; 20× magnification, scale bar is 50 μm. Quantification of (D) Sirius Red and (E) Masson's trichrome staining as a percentage of total area from eight pictures from each slice (n = 3‐4). Data are presented as means ± SEM; *P < .05 compared to control, ^#^ *P* < .05 compared to UUO

To determine the effect of the EP_1_ receptor antagonist SC‐19220 on extracellular matrix (ECM) deposition, we determined the expression of FN, αSMA, Collagen 1a1 and Collagen 3a1. As shown in Figure 2, gene expression of all four markers increased after UUO. However, we did not observe a statistically significant decline in transcript levels following SC‐19220 treatment. SC‐19220 markedly reduced UUO‐induced FN and αSMA protein expression, as shown by both WB (Figure 3A,B) and IHC (Figure 3C). In addition, UUO resulted in tubular dilation, which was partially blocked by EP_1_ receptor antagonism (Figure 4A). Moreover, we observed a clear increase in interstitial collagen deposition in UUO mice as shown by both Sirius red (Figure 4B) and Masson's trichrome (Figure 4C) staining, which was significantly reduced by treatment with SC‐19220 (Figure 4B‐E). Taken together, these results indicate that inhibition of the EP_1_ receptor can attenuate UUO‐induced fibrogenesis on the protein level in UUO mice.

### 2.3 Effect of EP_1_ inhibition on renal functional parameters in mice subjected to 7dUUO

To determine whether inhibition of the EP_1_ receptor would affect renal function in UUO mice, we evaluated kidney weight, creatinine, blood urea nitrogen (BUN) and major electrolytes. The results are summarized in Table 1. After 7 days, the bodyweight of the animals was unchanged, and the mice showed no signs of poor health. As expected, there was an increase in the weight of the left obstructed kidney of mice subjected to UUO when compared to sham‐operated mice. This effect was similar in mice exposed to SC‐19220. BUN was increased in mice subjected to UUO compared to sham‐operated animals. However, in mice treated with SC‐19220, BUN was significantly different from sham. Despite this finding, it must be noted that the BUN value after UUO was similar with and without SC‐19220. Plasma creatinine, sodium and potassium did not differ among the four groups (Table 1). Collectively, these results show that renal function and overall health was not affected by inhibition of the EP_1_ receptor.

**TABLE 1:** Physiological parameters of UUO mice

Groups	n	Bodyweight (g)	Obstructed‐kidney/body weight (mg/g)	Creatinine (μmol/L)	BUN (mmol/L)	Na (mmol/L)	K (mmol/L)
SHAM	10	19.3	5.76 ± 0.17	15.1 ± 1.1	5.4 ± 0.4	150 ± 0.7	4.7 ± 0.06
SHAM+EP_1_ anta.	10	20.9	5.42 ± 0.08	13.1 ± 1.1	6.8 ± 0.3*	150 ± 0.6	4.8 ± 0.09
UUO	10	20.5	6.79 ± 0.19*	17.7 ± 1.4	7.8 ± 0.3*	151 ± 0.5	4.4 ± 0.11
UUO+EP_1_ anta.	10	19.8	7.32 ± 0.29^#^	17.4 ± 1.7	7.4 ± 0.6^#^	149 ± 0.7	4.5 ± 0.09

Note

Values are presented as the mean ± SEM.

Abbreviations: BUN, blood urea nitrogen; K, potassium; Na, sodium.

* *P* < .05 compared to sham.

^#^ *P* < .05 compared to sham+EP_1_ antagonist.

### 2.4 Inhibition of the EP_1_ receptor ameliorates early and late stage fibrosis in human renal tissue

Next, we evaluated whether inhibition of the EP_1_ receptor could ameliorate fibrosis directly in human precision‐cut kidney slices (PCKS). To this end, human PCKS were incubated with transforming growth factor‐β (TGF‐β, 10 ng/mL) for 24 and 48 hours in the absence or presence of SC‐19220 (225 µM).

**FIGURE 5:**
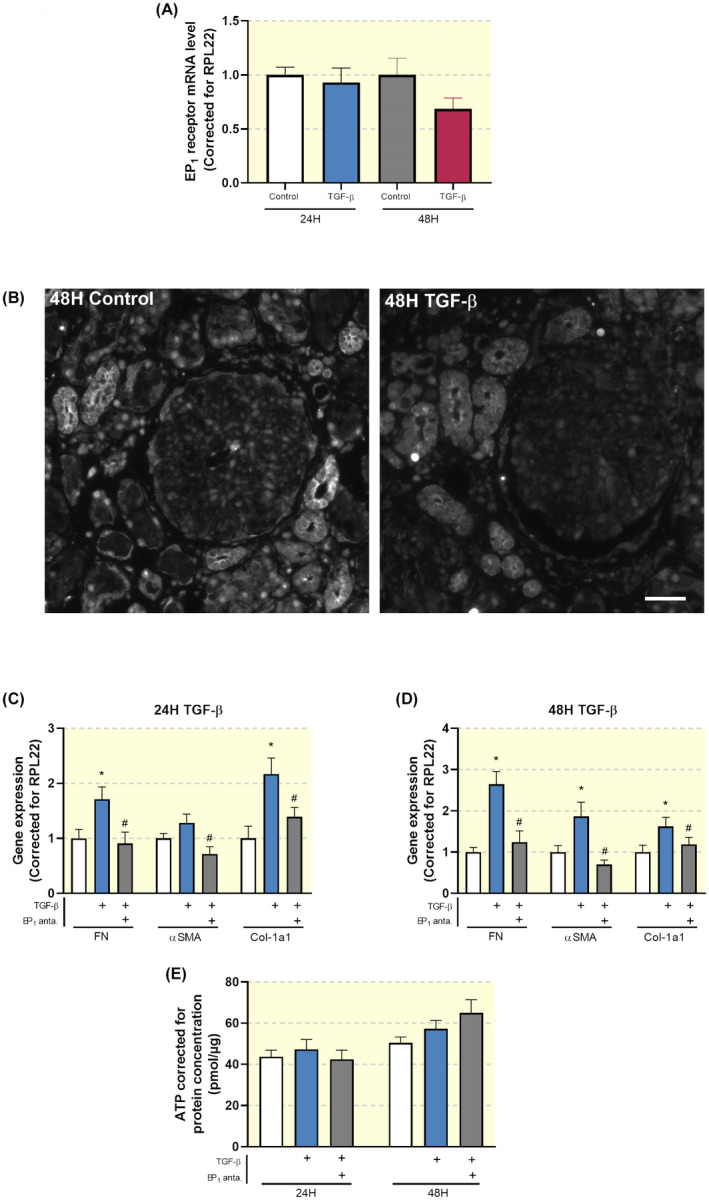
Inhibition of the EP_1_ receptor attenuates TGF‐β‐induced fibrosis in human precision‐cut kidney slices (PCKS). (A) EP_1_ receptor mRNA expression was studied by qPCR in human PCKS incubated with TGF‐β for 24 and 48 hours. Relative expression was calculated using the reference gene RPL22 (n = 10). (B) Representative immunofluorescent images of EP_1_ receptor expression in human PCKS. Scale bar is 50 μm. (C, D) Gene expression of fibronectin (FN), alpha‐smooth muscle actin (αSMA) and collagen 1a1 (Col‐1a1) was studied by qPCR in human PCKS incubated with TGF‐β (10 ng/mL) in the absence or presence of SC‐19220 (225 µM) for 24 and 48 hours, respectively. Relative expression was calculated using the reference gene RPL22 (n = 13). (E) Viability of human PCKS after treatment with TGF‐β in the absence or presence of SC‐19220, assessed by the ATP content of the slices (n = 13). Data are presented as means ± SEM; *P < .05 compared to control, ^#^ *P* < .05 compared to TGF‐β‐incubated human PCKS

We first investigated whether TGF‐β treatment could change the gene expression of the EP_1_ receptor in PCKS. As shown in Figure 5A, incubation of PCKS with TGF‐β up to 48 hours did not significantly affect the expression of the EP_1_ receptor. This finding was confirmed by visual inspections of immunofluorescent stainings where labelling intensity of the EP_1_ receptor in both TGF‐β and control samples seemed identical (Figure 5B).

**FIGURE 6:**
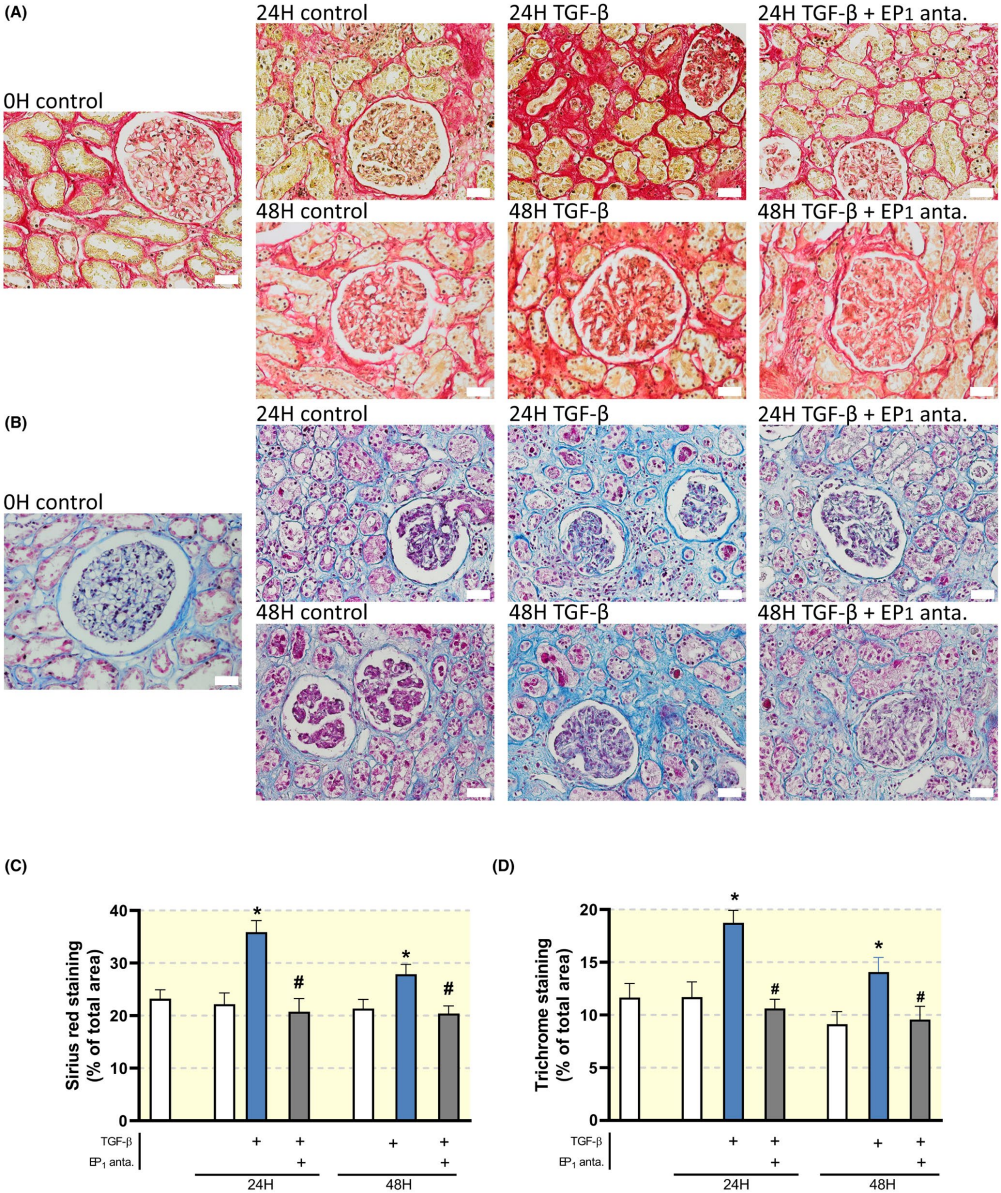
Inhibition of the EP_1_ receptor attenuates TGF‐β‐induced collagen deposition in human precision‐cut kidney slices (PCKS). Human PCKS were incubated with TGF‐β (10 ng/mL) in the absence or presence of SC‐19220 (225 µM) for 24 and 48 hours. Afterwards, renal fibrosis was assessed by IHC. Representative microscopy images of human PCKS following (A) Sirius red staining, (B) Masson's trichrome staining; 40× magnification, scale bar is 20 μm. Quantification of (C) Sirius Red and (D) Masson's Trichrome staining as a percentage of total area from five pictures from each slice. Data are presented as means ± SEM; *P < .05 compared to control, ^#^ *P* < .05 compared to TGF‐β‐incubated human PCKS

Exposure to TGF‐β induced fibrosis in human PCKS as illustrated by an increase in the gene expression of FN, αSMA and collagen 1a1 (Figure 5C,D). Inhibition of the EP_1_ receptor mitigated TGF‐β‐induced fibrogenesis, without affecting PCKS viability (Figure 5C‐E). We observed that the inhibitory effect of SC‐19220 on TGF‐β‐induced fibrogenesis was similar both in the presence and absence of indomethacin. To further examine the effect of EP_1_ receptor antagonism on collagen deposition in human PCKS, we performed Sirius red (Figure 6A) and Masson's trichrome staining (Figure 6B). Figure 6A‐D shows increased collagen deposition upon TGF‐β exposure, which could be attenuated by SC‐19220 treatment.

**FIGURE 7:**
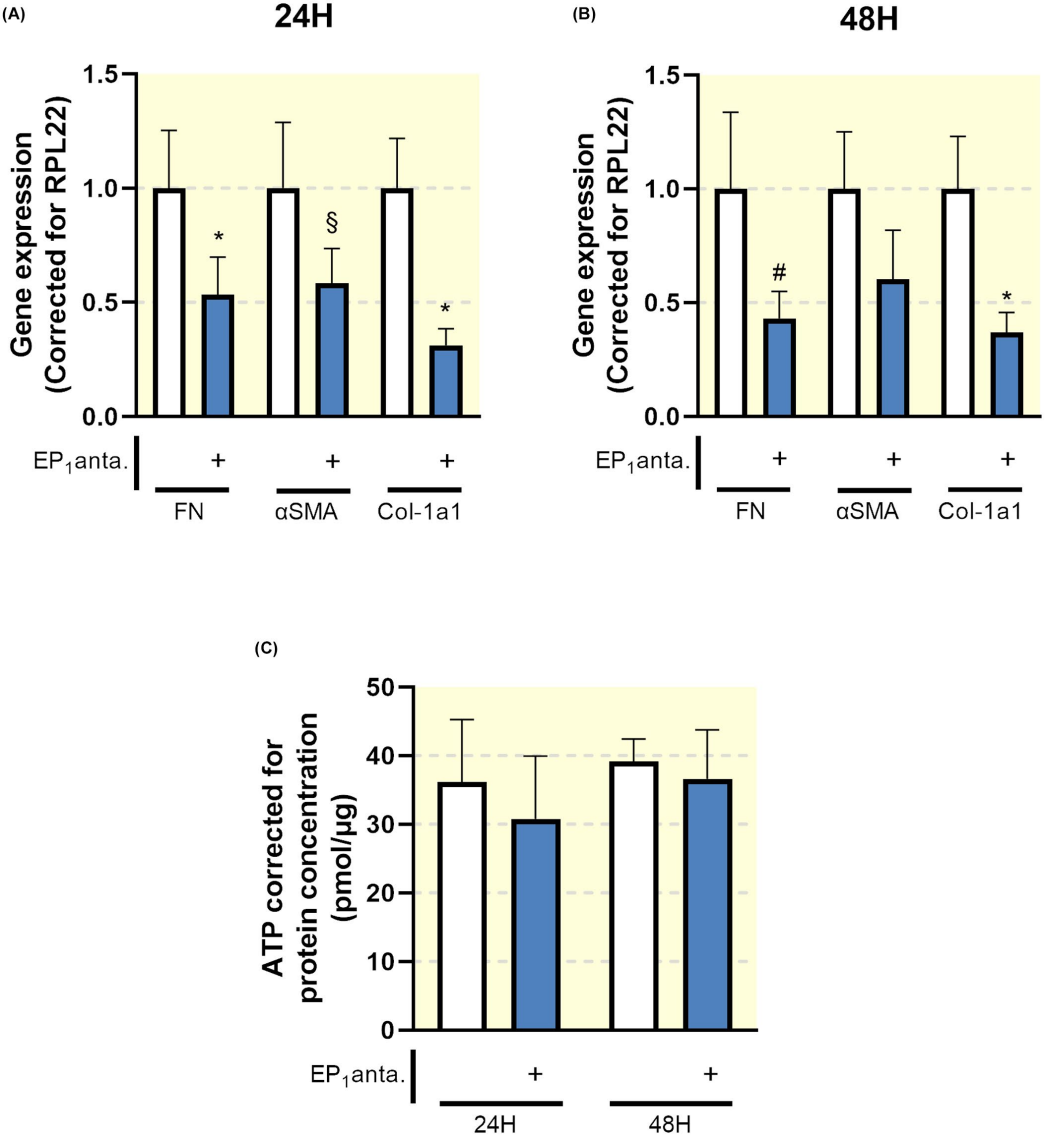
EP_1_ receptor antagonism attenuates late stage fibrosis in human precision‐cut kidney slices (PCKS). Human PCKS were incubated with TGF‐β (10 ng/mL) in the absence or presence of SC‐19220 (225 µM) for 24 and 48 hours. (A, B) Gene expression of fibronectin (FN), alpha‐smooth muscle actin (αSMA) and collagen 1a1 (Col‐1a1) was studied by qPCR in fibrotic slices incubated for 24 (A) and 48 hours (B). Relative expression was calculated using the reference gene RPL22 (n = 6). (C) Viability of PCKS after treatment with SC‐19220, assessed by the ATP content of the slices (n = 6). Data are presented as means ± SEM; *P < .05 compared to control. ^§^ *P* < .0507 compared to control. ^#^ *P* < .056 compared to control

Additionally, SC‐19220 treatment reduced the expression of FN, αSMA and collagen 1a1 in PCKS prepared from patients with established renal fibrosis (Figure 7). Taken together, these data indicate that antagonizing the EP_1_ receptor with SC‐19220 ameliorates early and late stage renal fibrosis in a translational model of human disease.

### 2.5 TGF‐β‐induced epithelial de‐differentiation is prevented by EP_1_ receptor antagonism in MDCK cells

**FIGURE 8:**
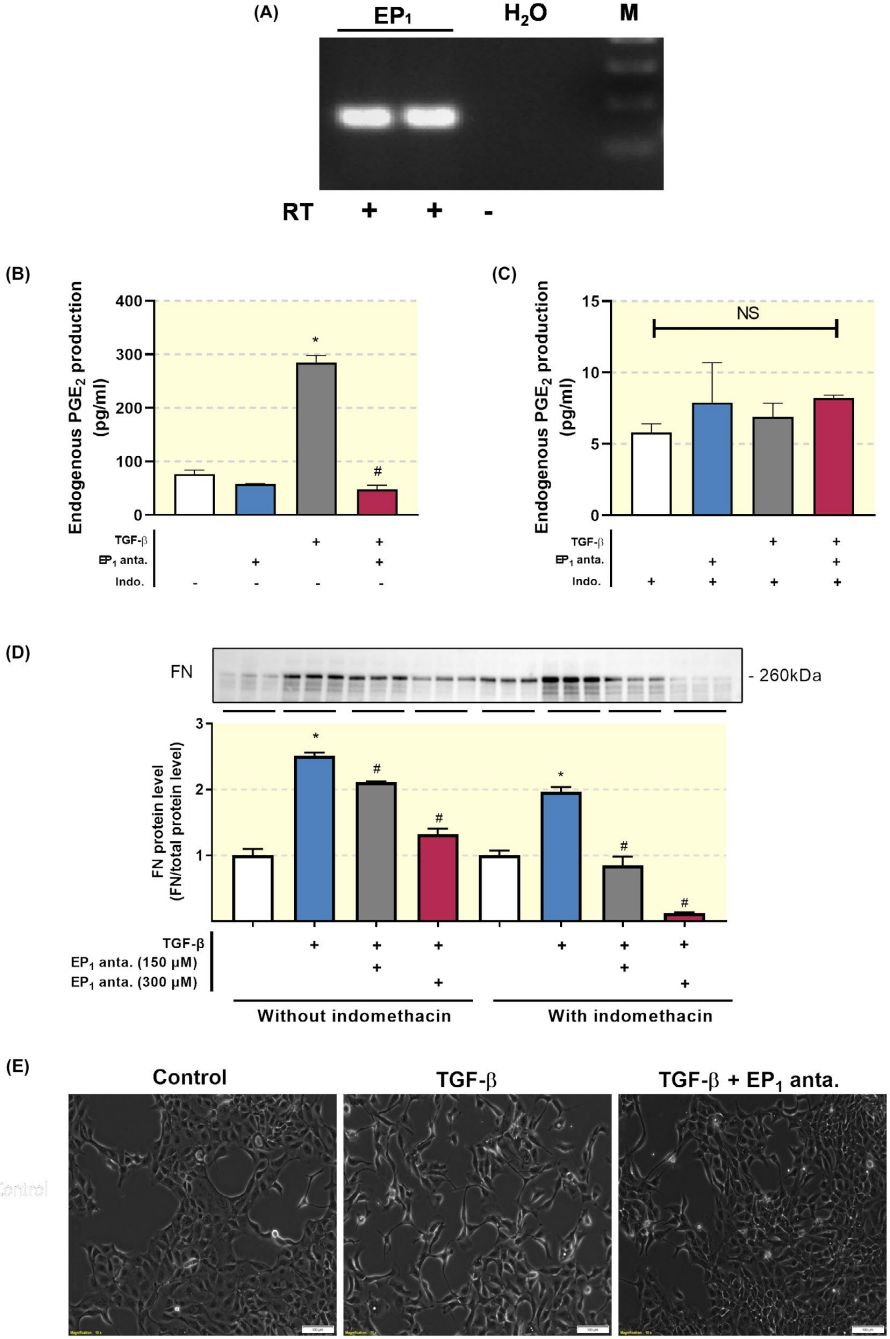
TGF‐β‐induced epithelial de‐differentiation is prevented by EP_1_ receptor antagonism in MDCK cells. (A) EP_1_‐receptor mRNA expression was studied in MDCK cells using RT‐PCR with (+) and without (−) reverse transcriptase (RT) enzyme. (B, C) MDCK cells were exposed to TGF‐β (10 ng/mL) treatment in the presence or absence of the EP_1_ antagonist SC‐19220 (225 µM) for 48 hours. PGE_2_ levels in culture media were measured with and without the non‐specific COX inhibitor indomethacin. Each bar represents the mean ± SEM of six experiments. (D) Western blot of fibronectin (FN) protein expression. Protein levels were calculated in relation to total protein. Each bar represents the mean ± SEM of three experiments. (E) Microscope images illustrating MDCK cell morphology. Scale bar is 100 μm. *P < .05 compared with control; ^#^ *P* < .05 compared with SC‐19220

Epithelial plasticity is an essential part of renal fibrogenesis.^21^ To mimic this process, we used TGF‐β to induce de‐differentiation of MDCK cells. Subsequently, we evaluated the impact of SC‐19220 on TGF‐β‐induced phenotypical changes. First, we confirmed the presence of the EP_1_ receptor in MDCK cells by RT‐PCR (Figure 8A). Next, we wanted to ensure that the effects of SC‐19220 did not interfere with endogenous PGE_2_ production. Therefore, PGE_2_ production was measured in MDCK cells in the presence or absence of the non‐specific COX inhibitor indomethacin to eliminate endogenous PGE_2_ production (Figure 8B,C). In the absence of indomethacin, endogenous PGE_2_ production was significantly increased when cells were exposed to TGF‐β. Surprisingly, this response was completely abolished when cells were treated with the EP_1_ receptor antagonist SC‐19220, suggesting an EP_1_ receptor‐dependent feed‐forward response of TGF‐β‐induced de novo prostaglandin synthesis. The addition of indomethacin markedly reduced endogenous PGE_2_ production in all groups, short‐circuiting both the effect of TGF‐β and SC‐19220 when using PGE_2_ production as the read out.

As shown in Figure 8D, exposure of MDCK cells to TGF‐β increased the protein expression of FN. This effect of TGF‐β was concentration‐dependently reduced by SC‐19220. Additionally, indomethacin potentiated the effect of SC‐19220, suggesting that other receptors than EP_1_ are involved in the TGF‐β‐dependent induction of FN expression. Moreover, in the presence of indomethacin, one can still observe a concentration‐dependent reduction of FN expression, suggesting that there still is sufficient baseline PGE_2_ production to support EP_1_ receptor activation. In addition, light microscopy revealed that treatment with TGF‐βinduced a phenotypic transition of MDCK cells, causing the cells to acquire a spindle‐like morphology (Figure 8E). Moreover, treatment of the cells with SC‐19220 reduced TGF‐β‐induced epithelial de‐differentiation (Figure 8E). These findings demonstrate that EP_1_ receptor antagonism can prevent the loss of epithelial characteristics.

### 2.6 Influence of the EP_1_ receptor on the intracellular Ca^2+^ ([Ca^2+^]_i_) response in MDCK cells

It has been suggested that the EP_1_ receptor can couple to both G_q_ and G_i_.^22^ It has previously been demonstrated that the EP_1_ receptor contributes to fibrogenesis in cardiac fibroblasts via a Ca^2+^‐dependent signalling pathway involving release from intracellular Ca^2+^ stores.^23^ Thus, one could speculate that inhibition of the EP_1_ receptor mitigates TGF‐β‐induced fibrosis by interfering with intracellular Ca^2+^ signalling. Therefore, we examined whether stimulating the EP_1_ receptor with 17‐phenyl trinor PGE_2_ could trigger a [Ca^2+^]_i_ response in MDCK cells.

**FIGURE 9:**
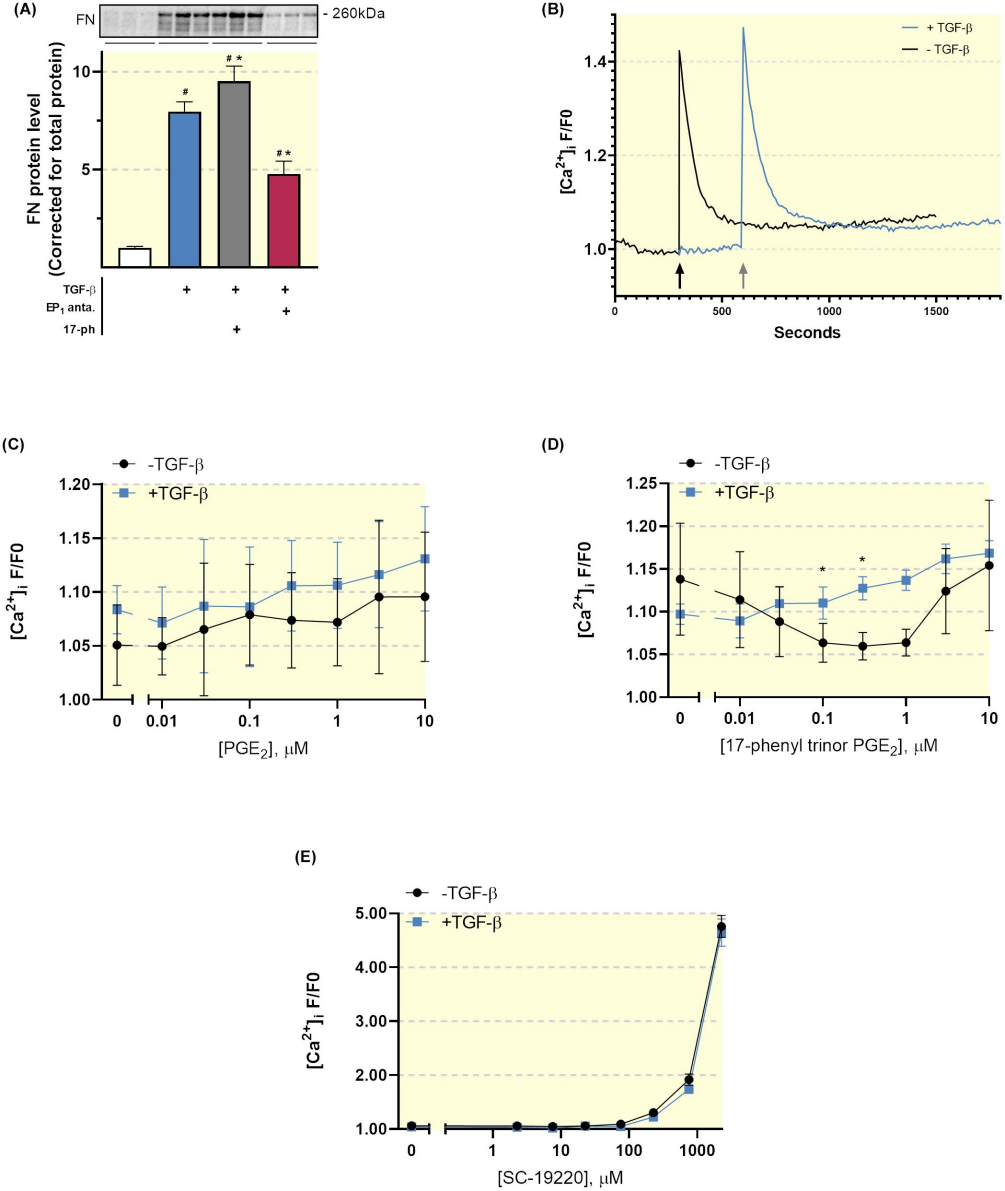
Anti‐fibrotic effect of SC‐19220 in MDCK cells does not involve [Ca2+]_i_ signalling. (A) MDCK cells were exposed to TGF‐β (10 ng/mL) treatment in the presence or absence of the EP_1_ antagonist SC‐19220 (225 µM) and EP_1_ agonist 17‐phenyl trinor PGE_2_ (1 µM) for 48 hours. Fibronectin (FN) protein expression was studied using western blotting. Protein levels were calculated in relation to total protein (n = 6). 17‐ph: 17‐phenyl trinor PGE_2_. Data are presented as mean ± SEM; *P < .05 compared to control, ^#^ *P* < .05 compared to TGF‐β. (B) Fluorescence trace of [Ca^2+^]_i_ as determined by Fluo‐4 AM in MDCK cells incubated with or without TGF‐β for 24 hours prior to the measurement. The arrows indicate when ATP was added during the measurement. Data is shown as F/F0. F/F0 values are reported as the mean of the five highest values at each given dose/treatment during the 20‐minute measurements. Fluo‐4 AM dose‐response curve of MDCK cells exposed to (C) PGE_2_, (D) 17‐phenyl trinor PGE_2_ and (E) SC‐19220 with or without TGF‐β pre‐treatment. *P < .05 compared to cells without TGF‐β pre‐treatment

Our results demonstrated that the EP_1_ receptor agonist 17‐phenyl trinor PGE_2_ potentiated TGF‐β‐induced fibrosis, as evaluated by FN protein levels, further supporting the notion that EP_1_ receptor activity stimulates fibrogenesis. Interestingly, the pro‐fibrotic effect of TGF‐β in combination with 17‐phenyl trinor PGE_2_ was markedly reduced by SC‐19220 treatment (Figure 9A).

Next, we measured the effect of SC‐19220 on the [Ca^2+^]_i_ response in MDCK cells in the absence or presence of TGF‐β. To this end, cells were loaded with the fluorescent Ca^2+^ probe Fluo‐4 AM. As positive control for cell responsiveness, we used ATP (100 µM). ATP induced a brisk, transient increase in [Ca^2+^]_i_, which is a trademark for G_q_ coupled IP_3_‐mediated Ca^2+^ release from intracellular Ca^2+^ stores mediated by the P2Y_2_ receptor. Pre‐incubation with TGF‐β increased the ATP‐induced [Ca^2+^]_i_ response (Figure 9B). Our data showed that neither PGE_2_ nor 17‐phenyl trinor PGE_2_ was able to elicit a [Ca^2+^]_i_ response resembling G_q_ activation in MDCK cells, irrespective of whether the cells were incubated with or without TGF‐β (Figure 9C,D). Thus, we were unable to evaluate the effect of SC‐19220 on EP_1_ activation with [Ca^2+^]_i_ as readout. However, we did ascertain whether SC‐19220 had any unexpected effects on [Ca^2+^]_i_. Our data shows that SC‐19220, at low, pharmacologically relevant concentrations, did not impact [Ca^2+^]_i_. However, at concentrations above 225 µM, SC‐19220 induced a massive increase in [Ca^2+^]_i_, which is sometimes observed at high concentrations of arachidonic acid derivatives (Figure 9E). Thus, it appears that, in our hands, the anti‐fibrotic effect of SC‐19220 does not involve [Ca^2+^]_i_ signalling.

### 2.7 EP_1_ receptor inhibition attenuates ERK1/2 signalling in MDCK cells

**FIGURE 10:**
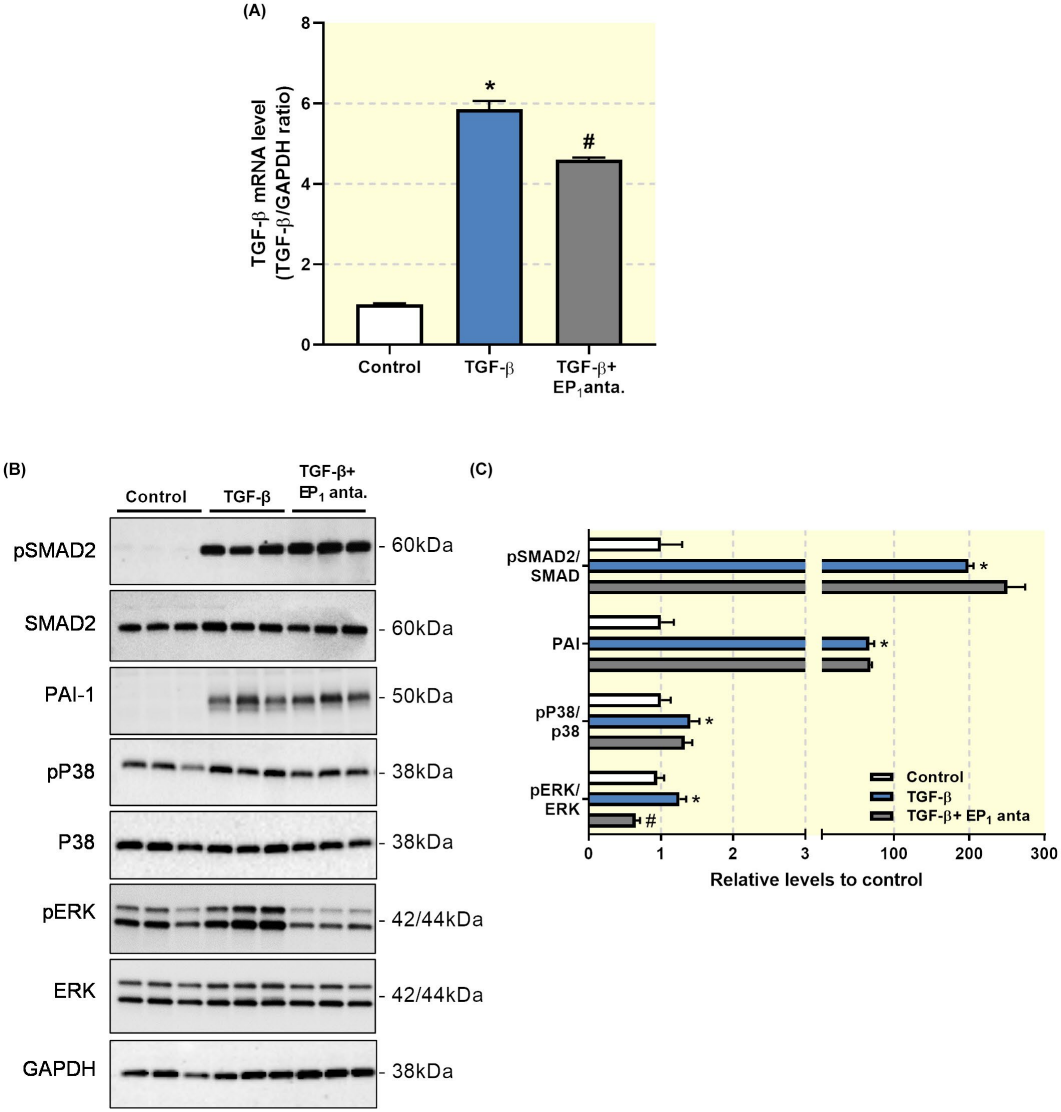
Anti‐fibrotic effect of SC‐19220 in MDCK cells involves ERK1/2 signalling. (A) MDCK cells were exposed to TGF‐β (10 ng/mL) treatment in the presence or absence of the EP_1_ antagonist SC‐19220 (225 µM) for 48 hours. TGF‐β mRNA expression was studied by qPCR. Relative expression was calculated using the reference gene GAPDH (n = 6). (B) Representative Western Blots of proteins related to SMAD and MAPK signalling pathways. (C) Quantification of protein expression relative to control (n = 6). Data are presented as means ± SEM *P < .05 compared to control, ^#^ *P* < .05 compared to TGF‐β

In order to elucidate the mechanisms of action of SC‐19220, we explored its impact on the TGF‐β signalling pathway, which plays an important role in fibrogenesis. As shown in Figure 10A, the EP_1_ receptor antagonist reduced TGF‐β mRNA expression in MDCK cells. Next, we studied the expression of plasminogen activation inhibitor 1 (PAI‐1) and the activation of Smad2, which are both major downstream elements in TGF‐β signalling. TGF‐β induced Smad2 phosphorylation and increased the expression of PAI‐1, which was not impacted by SC‐19220 treatment (Figure 10B,C). Thus, it appears that the mechanism of action of SC‐19220 in MDCK cells does not involve the Smad or PAI pathway (Figure 10B,C). It has previously been shown that TGF‐β can activate MAPK pathways in MCDK cells, which is an important intracellular signalling pathway in renal fibrosis.^24^ So, we examined whether the anti‐fibrotic effect of SC‐19220 could be mediated via the MAPK signalling pathway. Our results revealed that inhibition of the EP_1_ receptor suppressed TGF‐β‐induced phosphorylation of ERK1/2 whereas no effect was observed on p38 (Figure 10B,C). Taken together, these results indicate that EP_1_ receptor antagonism with SC‐19220 mitigates fibrogenesis by reducing ERK1/2 signalling.

### 2.8 Inhibition of the EP_1_ receptor reduces matrix formation

**FIGURE 11:**
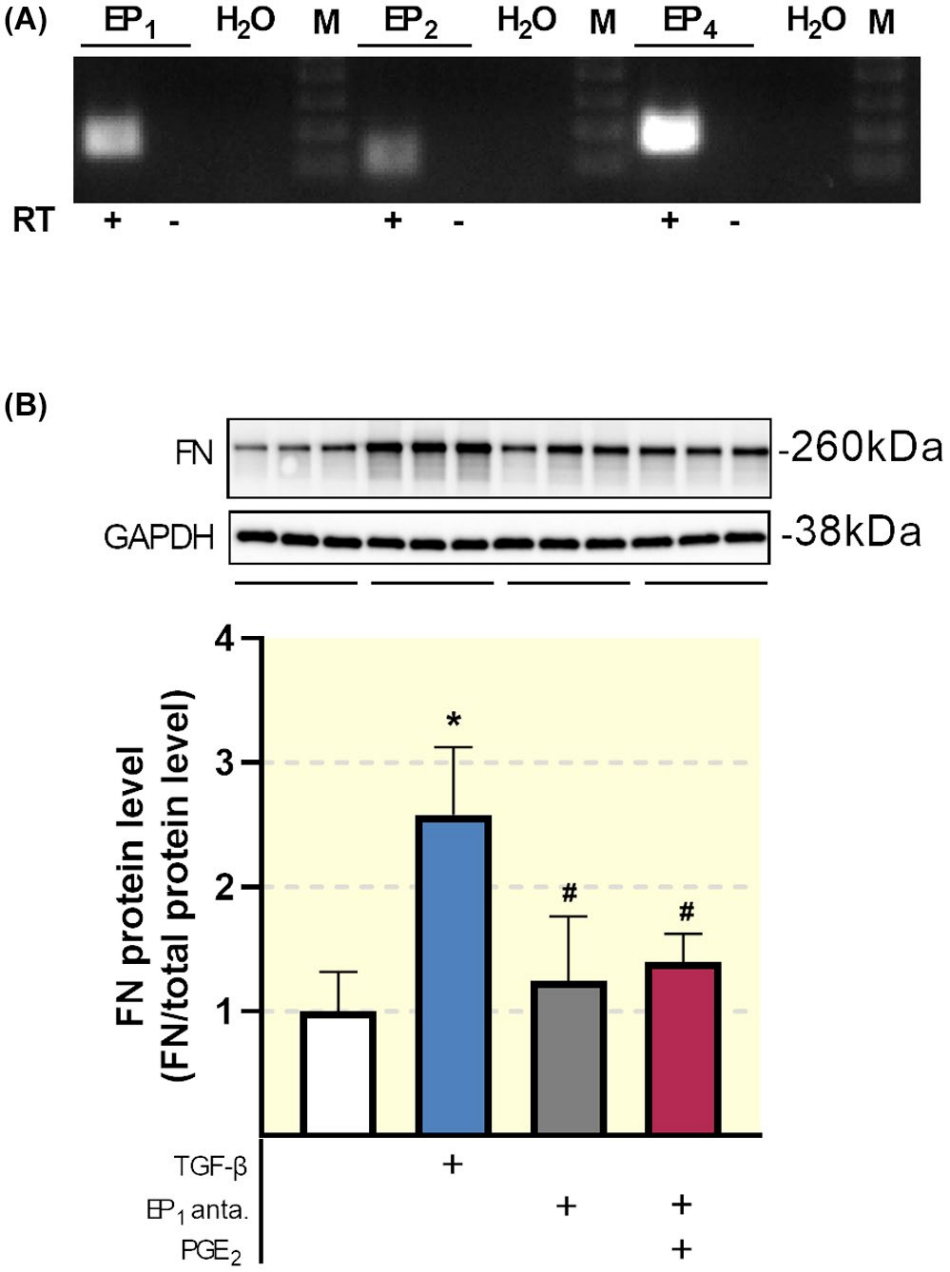
Impact of SC‐19220 treatment in human renal fibroblasts. (A) EP receptor mRNA expression was studied in HRFs using RT‐PCR with (+) and without (−) reverse transcriptase (RT) enzyme. (B) HRFs were exposed to TGF‐β (10 ng/mL) in the presence or absence of the EP_1_ antagonist SC‐19220 (225 µM) and PGE_2_ (1 µM) for 48 hours. Fibronectin (FN) protein expression was studied using Western Blotting. Protein levels were calculated in relation to total protein (n = 6). Data are presented as means ± SEM; ^#^ *P* < .05 compared to TGF‐β

Lastly, we set out to confirm our observations in human renal fibroblasts—key cellular players in the fibrotic process. As shown in Figure 11A, EP_1_, EP_2_ and EP_4_ are all expressed in HRFs. Moreover, exposure to TGF‐β markedly increased the protein expression of FN, which was mitigated by SC‐19220 treatment (Figure 11B). In addition, the anti‐fibrotic effect of EP_1_ antagonism was not affected by PGE_2_.

## 3 DISCUSSION

Preclinical studies have identified a number of approaches to either stop or reverse the progression of renal fibrosis.^25^ However, none of these treatments have been successfully implemented in the clinic.^26^ The availability of good translational models of human fibrosis, which can be used to directly validate preclinical findings, would improve drug development. In this study, we investigated the anti‐fibrotic properties of a specific EP_1_ receptor antagonist with a translational approach utilizing well‐established in vitro and in vivo fibrosis models as well as an ex vivo human model of renal fibrosis, namely human (fibrotic) PCKS. Thus, we were able to validate our findings directly in a human model. By using these models, we show that antagonizing the EP_1_ receptor has considerable anti‐fibrotic effects.

In the current study, EP_1_ antagonism alleviates kidney fibrogenesis in MDCK cells, HRFs, UUO mice as well as in human PCKS. To our knowledge, our work is the first to confirm that pharmacological inhibition of the EP_1_ receptor using SC‐19220 prevents the progression of fibrosis in a human renal fibrosis model. We and others have previously confirmed that other EP receptors, including EP_2_ and EP_4_ play significant roles in preventing kidney fibrosis.^15^, ^27^ Recently, Jensen et al showed that activation of the EP_2_ receptor with butaprost mitigates fibrogenesis in human PCKS,^14^ indicating that targeting the EP_2_ receptor also directly prevents renal fibrosis in human kidney slices.

In the clinic, renal fibrosis is often diagnosed when the disease is in its final stages, making treatment even more difficult. Here, we show that EP_1_ receptor antagonism might also be beneficial for the treatment of established fibrosis, further supporting the notion that the EP_1_ receptor is a clinically relevant therapeutic target.

Several in vitro studies have demonstrated that EP_1_ receptor activation can cause detrimental responses in different renal cell types, including mesangial cells, proximal tubule cells and podocytes.^16^, ^28^, ^29^ In addition, in vivo studies have shown that a lack of the EP_1_ receptor or EP_1_ antagonism protects against hyperfiltration, albuminuria, and reduces injury/fibrotic markers in spontaneously hypertensive rats^17^, ^30^ and in diabetic mice models.^16^ Conjointly, these data suggest that the EP_1_ receptor mediates many pathologic effects in different kidney diseases, highlighting the importance of this receptor in renal diseases. Thus, the anti‐fibrotic effect of EP_1_ antagonism shown here corroborates previous work.

To eliminate the possibility that endogenous prostaglandins elicited the observed anti‐fibrotic effects via the EP_2_ or the EP_4_ receptor, we performed both our in vitro and ex vivo experiments in the absence and presence of the non‐specific COX inhibitor, indomethacin. MDCK cells exposed to TGF‐β in the absence of indomethacin showed dramatically increased levels of PGE_2_, in line with previous studies showing autoamplification of PGE_2_ through the EP_1_ receptor.^31^ Moreover, indomethacin lowered endogenous PGE_2_ production considerably; however, there is still sufficient baseline PGE_2_ production to support EP_1_ receptor activation. Therefore, the anti‐fibrotic effect of SC‐19220 might be because of its impact on the EP_1_ receptor, illustrating that the PGE_2_ receptors are promising therapeutic targets for the treatment of fibrosis, in line with the study of Jensen and colleagues.^14^

Based on our work, and that of others, it is clear that PGE_2_ signalling can be pro‐ or anti‐fibrotic depending on which receptor is activated.^16^, ^29^, ^32^, ^33^ Our results did not reveal how EP_1_ receptor antagonism impacted signalling via EP_2_ and EP_4_ during fibrogenesis. In order to fully unravel the role of PGE_2_ in human renal fibrosis, future studies are needed that can show which of the four PGE_2_ receptors is dominant during the fibrotic process. It is highly likely that the net effect of PGE_2_ on fibrosis will depend greatly on disease aetiology as well as cell‐ and tissue‐specific expression of the PGE_2_ receptors. Thus, even though our study provides another piece of the puzzle, the complex and dual role of PGE_2_ in renal fibrosis requires further scrutiny.

Stimulation of the EP_1_ receptor is known to increase intracellular Ca^2+^ concentration by a G_q_ dependent IP_3_ mobilization.^34^, ^35^ As seen in Figure 9, we were unable to elicit abrupt G_q_ dependent increases in [Ca^2+^]_i_ with neither PGE_2_ nor 17‐phenyl trinor PGE_2_. Together, our results indicated that the anti‐fibrotic effect of SC‐19220 is independent of [Ca^2+^]_i_ signalling, suggesting that other signalling pathways must be involved. Previous studies have demonstrated that TGF‐β can induce phenotypical changes via Smad‐2/3, PAI‐1, ERK1/2 and p38 MAPK signalling pathways in MDCK cells.^24^, ^36^ In line with this, our data revealed that TGF‐β stimulates phosphorylation of Smad2, ERK1/2 and p38 MAPK as well as induces PAI‐1 expression. SC‐19220 clearly inhibited the phosphorylation of ERK1/2 but had no effect on the phosphorylation of Smad2 and p38 MAPK nor on the expression of PAI‐1. This observation is in line with the study by Chen et al, showing that TGF‐β‐induced activation of the ERK1/2 MAPK pathway can be alleviated by EP_1_ knockdown and stimulated by EP_1_ expression in mesangial cells.^28^ These findings support the notion that the EP_1_ receptor might contribute to fibrogenesis via activation of the ERK1/2 signalling pathway.

In this study, we observed that SC‐19220 mitigated TGF‐β‐induced phenotypical changes in MDCK cells. The loss of epithelial characteristics upon TGF‐β exposure is well‐documented and is linked to the progression of renal fibrosis.^37^ For decades, it was believed that epithelial cells could convert to collagen‐producing myofibroblasts via a process called epithelial‐to‐mesenchymal transition. However, lineage‐tracing studies and single‐cell RNA sequencing has revealed that only 1%‐5% of de‐differentiated epithelial cells ultimately contribute to ECM production.^38^, ^39^ Nevertheless, the observed phenotypical changes might reflect a metabolic reprogramming, especially related to fatty acid oxidation, which can contribute to fibrogenesis.^37^, ^39^ Further studies are needed to reveal whether EP_1_ receptor antagonism can indeed restore fatty acid metabolism in TGF‐β exposed cells.

Gene and protein expression of the EP_1_ receptor does not seem to be affected after 7 days of UUO. This is consistent with a previous study by Sun et al, showing that seven days of UUO in mice did not change the expression of the EP_1_ receptor either at the mRNA or protein level.^40^ However, we have previously demonstrated that UUO increased EP_1_ receptor mRNA expression in wildtype and COX‐2 KO mice.^9^ In this study, we used another strain of mice, possibly altering the phenotype for this particular outcome. Consistent with previous studies, the EP_1_ receptor was mainly found in cells of the collecting ducts^41^ and TAL.^42^ In addition, the EP_1_ receptor was also detected in glomeruli.^43^, ^44^

A limitation of this study is that we administered SC‐19220 via IP injection, whereas oral delivery of an EP_1_ receptor antagonist would most likely be needed for treatment in humans. However, it is generally considered that the pharmacokinetics of small molecular drugs administered IP are fairly similar to those seen after oral administration, because the primary route of absorption is into the mesenteric vessels, which drain into the portal vein and pass through the liver, thus the compounds are subjected to first pass metabolism.^45^, ^46^ Moreover, from a technical point of view, IP delivery is easy, reproducible, ensures therapeutic bioavailability, allows for repeated treatment and is generally safer and less stressful for the animals as compared to other delivery methods. Thus, we believe that this route of administration is justifiable for preclinical drug efficacy studies. Moreover, most preclinical studies only use cell and animal models. By using human (fibrotic) kidney slices we have already greatly improved the clinical translatability of our findings.

In conclusion, this study indicates that the EP_1_ receptor might be regarded as a potential target for the treatment of renal fibrosis. The present study provides strong evidence that the effect of the EP_1_ antagonist SC‐19220 may translate to clinical care since its effects on fibrosis is demonstrated in both UUO mice as well as human kidney slices.

## 4 MATERIALS AND METHODS

### 4.1 Ethics statement

The procedures described below were performed in concordance with the Danish national guidelines for animal care and the published guidelines of the National Institutes of Health and approved by the institute's local committee according to the licenses for the use of experimental animals issued by the Danish Ministry of Justice (Approval number: 2015‐15‐0201‐00658). The use of human tissue for the preparation of PCKS was approved by the Central Denmark Region Committees on Biomedical Research Ethics (Approval number: 1‐10‐72‐211‐17) and the Danish Data Protection Agency. All participants gave written informed consent.

### 4.2 Experimental animals and surgical procedures

Experiments were performed using male C57BL/6 mice, 7‐8 weeks of age and weighing 20.1 ± 1.9 g (Janvier Labs, Le Genest‐Saint‐Isle, France). Animals were housed with a 12 h:12 h light‐dark cycle, a temperature of 21 ± 2°C and a humidity of 55 ± 2%. Animals had ad libitum access to standard rodent chow (Altromin, Lage, Germany) and tap water. Animals were allowed to acclimatize 7 days before surgery. A preliminary dose‐response study, using SC‐19220 (5, 10 and 25 mg/kg, Cayman Chemical, Michigan, USA) was performed on 3‐4 animals per group. Since 25 mg/kg was most effective in lowering the expression of αSMA and FN in the pilot study (Figure S1), this dose was validated in a larger cohort. In the subsequent study, C57BL/6 male mice were allocated into the following experimental groups: SHAM operated mice (n = 14, 10 kidneys were used for qPCR and WB, and 4 kidneys were used for IHC), SHAM operated mice treated with 25 mg/kg of SC‐19220 (n = 10, 10 kidneys were used for qPCR and WB), UUO (n = 14, 10 kidneys were used for qPCR and WB and 4 kidneys were used for IHC) and UUO operated mice treated with 25 mg/kg once a day (n = 14, 10 kidneys were used for qPCR and WB and 4 kidneys were used for IHC).

On the day of surgery, mice were anaesthetized with 2% Sevoflurane (Abbott Scandinavia AB, Solna, Sweden) mixed with atmospheric air at 2 L/min, and injected with buprenorphine (Temgesic, Indivior UK Limited, Berkshire, UK). A midline incision was made and the left ureter was located and occluded with a 6‐0 silk ligature. The EP_1_ receptor antagonist SC‐19220 was diluted in saline and administered once daily via intraperitoneal injection starting at the day of the surgery. Buprenorphine was added to the drinking water to maintain analgesia for 48 hours post‐surgery. After 7 days of UUO, blood samples were collected by cardiac puncture, the kidneys were collected, and the mice were sacrificed by cervical dislocation. Biochemical analysis of blood samples was performed on a Roche Cobas 6000 analyzer (Roche Diagnostic, Rotkreuz, Switzerland) and creatinine levels were determined using a creatinine assay kit (Sigma‐Aldrich, Missouri, USA), according to the manufacturer's instructions.

### 4.3 Human precision‐cut kidney slices

PCKS were prepared from functional (eGFR >60 mL/min/1.73 cm^2^) and macroscopically healthy renal cortical tissue obtained from both male and female patients following tumour nephrectomies as described previously.^14^ In addition, PCKS were also prepared from human fibrotic kidneys (see Table 2 for patient demographics). In short, tissue samples were obtained using a 6 mm biopsy punch (Kai Medical, Japan), and slices were prepared in ice‐cold Krebs‐Henseleit buffer (25 mM D‐glucose, 25 mM NaHCO_3_, 10 mM HEPES, saturated with 95% O_2_ and 5% CO_2_) using the Krumdieck Tissue slicer. Slices were cultured in William's medium E containing GlutaMAX, 10 mg/mL ciprofloxacin and 2.7 g/L D‐(+)‐Glucose in an 80% O_2_, 5% CO_2_ atmosphere at 37°C. The slices were kept in constant moderate motion and the medium was replaced every 24 hours. ATP content was used as a viability marker and was determined using the ATP Colorimetric/Fluorometric Assay Kit (Sigma‐Aldrich, St. Louis, MO, USA), according to the supplied instructions.

**TABLE 2:** Patient demographics

Parameter	Healthy renal tissue	Fibrotic renal tissue
Gender (% male)	77	66
Age (in years)	62 ± 15	43 ± 23
BMI	25.9 ± 8.9	26.8 ± 4.7
eGFR (mL/min/1.73 m^2^)^a^	71.7 ± 8.8	62 ± 19.5
Renography (%)	NA	8.8 ± 3.4
Ischemia time (min)	44.5 ± 18.6	32 ± 19.5
Number patients	13	6

Note

Values are presented as the mean ± standard deviation.

Abbreviations: BMI, body mass index; eGFR, estimated glomerular filtration rate; NA, not available.

^a^ Calculated using the Modification of Diet in Renal Disease (MDRD) formula.

### 4.4 MDCK and human renal fibroblast cell cultures

Madin‐Darby Canine Kidney (MDCK) cells and primary human renal fibroblast (HRFs; AU009F DV Biologics Lot# 061314CA male) were used to investigate the anti‐fibrotic potency of SC‐19220 (Sigma Aldrich, St. Louis, MO, USA) in vitro. HRFs were kindly provided by Prof. RA Bank, University of Groningen, the Netherlands. The cells were cultured in Dulbecco's Modified Eagle Medium (DMEM) with 10% fetal bovine serum and 1% penicillin/streptomycin. The cells were kept at 37°C in a 5% CO_2_ atmosphere and grown to 80% confluence. The appropriate dose of SC‐19220 was titrated in a concentration‐response experiment, using 150‐300 µM. Here, WB analysis of FN confirmed that SC‐19220 elicited a concentration‐dependent antifibrotic effect, so we decided to use a concentration of 225 μM SC‐19220 for the forthcoming experiments. Accordingly, experiments consisted of 48 hours of TGF‐β stimulation, and subsequent treatment with SC‐19220 (225 µM, Sigma‐Aldrich, St. Louis, MO, USA), 17‐phenyl trinor PGE_2_ (1 μM, Cayman Chemical, MI, USA) or PGE_2_ (1 μM, Sigma Aldrich, St. Louis, MO, USA). SC‐19220 and 17‐phenyl trinor PGE_2_ were administered 30 minutes prior to the addition of TGF‐β. Additionally, experiments were performed in the absence and presence of indomethacin (5 µM, Sigma‐Aldrich, St. Louis, MO, USA) to evaluate the effects of endogenous PGE_2_. Furthermore, media was collected and endogenous PGE_2_ production was measured with a PGE_2_ ELISA kit (Abcam, Cambridge, UK), according to the manufacturer's instructions.

### 4.5 Measurements of [Ca^2+^]_i_ in MCDK cells

MDCK cells were cultured in a transparent 96‐well plate, either in the presence or absence of TGF‐β (10 ng/mL) for 24 hours. Afterwards, MDCK cells were washed using HEPES buffered salt solution (HBS) and loaded with 5 µM Fluo‐4 AM for 60 minutes dissolved in HBS plus probenecid (5 mM). The cells were then thoroughly washed in HBS and baseline measurements were performed. Afterwards, MDCK cells were exposed to either PGE_2_, the EP_1_ agonist 7‐phenyl‐trinor PGE_2_, or the EP_1_ antagonist SC‐19220 in increasing concentrations. ATP (10 and 100 µM) was used as a positive control and HBS as a negative control. The experiments were carried out as a 20 minutes time lapse in a flourescence plate reader (Mithras LB 940, Berthold Technologies, Bad Wildbad, Germany). The cells were excited at 488 nm and emission collected >510 nm, with a frequency of 8‐15 measurements per minute depending on the number of wells included in the given experiment. Changes in [Ca^2+^]_i_ were shown as F/F_0_ ratio, where F_0_ is determined as baseline Fluo‐4 AM fluorescence before addition of test‐compounds (5 observations).

### 4.6 Western blotting

Total protein from mouse cortex was extracted using RIPA buffer including phosphatase inhibitor 2 and 3 (Sigma‐Aldrich, St. Louis, MO, USA) and a protease inhibitor cocktail tablet (Roche Diagnostic, Rotkreuz, Switzerland) and total protein from cell experiments was extracted using M‐PER including 2% SDS and DTT. Protein was then separated on a 12% Criterion TGX Stain‐free gel and proteins were transferred to a nitrocellulose membrane, which was blocked with 5% skimmed milk in PBS‐Tween. The membrane was washed in PBS‐Tween and incubated with specific primary antibodies (see Table 3 for target and dilution). Next, the membrane was washed with PBS‐Tween and incubated with the appropriate secondary antibody. Finally, the membrane was incubated with the detection reagent ECL‐Prime (GE‐healthcare, Chicago, IL, USA) and processed in the Western Blot Imager (ChemiDoc MP, Bio‐Rad, CA, USA). Detected protein was normalized against total protein levels.^47^

**TABLE 3:** Primary antibodies used for western blotting and immunohistochemistry/fluorescence

Target	Catalogue number	Company	Species	Dilution
EP_1_	APR.063	Alamone	Rabbit	1:250
AQP2	Sc‐9880	Santa Cruz	Goat	1:200
THP	AB733	Millipore	Sheep	1:200
FN	AB2413	Abcam	Rabbit	1:1000
αSMA	M0851	Dako	Mouse	1:500
pSMAD2	3108S	Cell Signaling	Rabbit	1:500
SMAD2	5339S	Cell Signaling	Rabbit	1:500
PAI‐1	PA5‐79980	Invitrogen	Rabbit	1:500
pP38	#4631	Cell Signaling	Rabbit	1:1000
P38	#9212	Cell Signaling	Rabbit	1:1000
pERK	#4370	Cell Signaling	Rabbit	1:500
ERK	#9102	Cell Signaling	Rabbit	1:500
GAPDH	#2118	Cell Signaling	Rabbit	1:1000

### 4.7 qPCR and RT‐PCR

RNA from cortical tissue and PCKS was isolated using the NucleoSpin RNA II mini kit (Macherey Nagel, Nordrhein‐Westfalen, Germany), while TRIzol Reagent (Life Technologies, Thermo Fisher Scientific, MA, USA) was used to isolate RNA from MDCK cells and HRFs. The RNA concentration was measured with spectrophotometry and samples were stored at −80°C until use. cDNA was synthesized using the RevertAid First Strand Synthesis Kit #K1622 (Thermo Fisher Scientific, MA, USA). Brilliant SYBR Green qPCR master Mix (Thermo Fisher Scientific, MA, USA), forward and reverse primers (Table 4) specific to the gene of interest were added and qPCR was carried out.

**TABLE 4:** Primers used for qPCR

Target gene	Species	Direction	Sequence
EP_1_	Mouse	Forward: Reverse:	5′‐CGGCATTAGTGTGCAATACG‐3′ 5′‐TGGCTGAAGTGATGGATGAG‐3′
EP_2_	Mouse	Forward: Reverse:	5′‐ATGCTCCTGCTGCTTATCGT‐3′ 5′‐AGGGCCTCTTAGGCTACTGC‐3′
EP_4_	Mouse	Forward: Reverse:	5′‐CCATCGCCACATACATGAAG‐3′ 5′‐TGCATAGATGGCGAAGAGTG‐3′
FN	Mouse	Forward: Reverse:	5′‐AATGGAAAAGGGGAATGGAC‐3′ 5′‐CTCGGTTGTCCTTCTTGCTC‐3′
18S	Mouse	Forward: Reverse:	5′‐TGTGGTGTTGAGGAAAGCAG‐3′ 5′‐TCCCATCCTTCACATCCTTC‐3′
Col 1a1	Mouse	Forward: Reverse:	5′‐CACCCTCAAGAGCCTGAGTC‐3′ 5′‐ACTCTCCGCTCTTCCAGTCA‐3′
Col 3a1	Mouse	Forward: Reverse:	5′‐GCACAGCAGTCCAACGTAGA‐3′ 5′‐TCTCCAAATGGGATCTCTGG‐3′
αSMA	Mouse	Forward: Reverse:	5′‐CTGACAGAGGCACCACTGAA‐3′ 5′‐CATCTCCAGAGTCCAGCACA‐3′
FN	Human	Forward: Reverse:	5′‐CAGTGGGAGACCTCGAGAAG‐3′ 5′‐GTCCCTCGGAACATCAGAAA‐3′
Col 1a1	Human	Forward: Reverse:	5′‐CCTGGATGCCATCAAAGTCT‐3′ 5′‐AATCCATCGGTCATGCTCTC‐3′
αSMA	Human	Forward: Reverse:	5′‐ACTGGGACGACATGGAAAAG‐3′ 5′‐TACATGGCTGGGACATTGAA‐3′
RPL‐22	Human	Forward: Reverse:	5′‐GGAGCAAGAGCAAGATCACC‐3′ 5′‐TGTTAGCAACTACGCGCAAC‐3′
EP_1_	Human	Forward: Reverse:	5′‐TTGTCGGTATCATGGTGGTG‐3′ 5′‐ATGTACACCCAAGGGTCCAG‐3′
EP_2_	Human	Forward: Reverse:	5′‐CCACCTCATTCTCCTGGCTA‐3′ 5′‐TTCCTTTCGGGAAGAGGTTT‐3′
EP_4_	Human	Forward: Reverse:	5′‐GACCTGTTGGGCACTTTGTT‐3′ 5′‐AGGTAGCGCTCGACACTCAT‐3′
TGF‐β	Dog	Forward: Reverse:	5′‐AAGAAAAGTCCGCACAGCAT‐3′ 5′‐GCTGCTCCGCTTTTAACTTG‐3′
GAPDH	Dog	Forward: Reverse:	5′‐AACATCATCCCTGCTTCCAC‐3′ 5′‐GGCAGGTCAGATCCACAACT‐3′
EP_1_	Dog	Forward: Reverse:	5′‐CTGTGAGCCTCTGCTCCTG‐3′ 5′‐GACAGCCAGCACCACTAACA‐3′

To confirm the expression of the EP receptors in MDCK cells and HRFs, RT‐PCR was performed either in the presence (+) or absence (−) of reverse transcriptase. To visualize the PCR product, electrophoresis was performed using a 1% agarose gel including the Genruler DNA marker (Invitrogen, CA, USA) and images were obtained on an Azure c200 gel imaging workstation.

### 4.8 Perfusion fixation and immunolabelling

For the preparation of tissue slices for histological analysis and IHC, we performed whole animal perfusion with subsequent immersion fixation (renal tissue) or immersion fixation (PCKS). Kidneys were fixed by perfusion via the left ventricle using 4% paraformaldehyde (PFA). Afterwards, the kidney was removed and immersed in 4% PFA for one hour, rinsed with PBS and dehydrated in a series of alcohol and embedded in paraffin. Tissue sections (2 and 5 μm) were deparaffinized, rehydrated and rinsed. Then, they were blocked with 1.5 mL 35% hydrogen peroxide (H_2_O_2_) in methanol for 30 minutes. For epitope retrieval, sections were boiled in TEG buffer (1 mM Tris, 0.5 mM ETA, pH of 9.0) then left to cool and blocked with 50 mM NH_4_Cl in PBS. Sections were then incubated with primary antibodies (EP_1_ receptor and FN for immunoperoxidase, see Table 3 for target and dilution) diluted in PBS containing 0.1% BSA and 0.3% Triton X100 for 1 hour at room temperature in a humidity chamber, followed by overnight incubation at 4°C. The sections were rinsed three times with PBS containing 0.1% BSA, 0.05% saponin and 0.2% gelatine followed by incubation with a secondary antibody (P448, diluted 1:300 in washing solution) for one hour at room temperature. Afterwards, sections were rinsed three times with washing solution and incubated with 3,3'diaminobenzidine tetrachloride (DAB) dissolved in water containing 0.1% H_2_O_2_ to visualize the sites of antibody‐antigen reactions. Light microscopy was carried out with an Olympus BX50 light microscope and images were processed using CellSens imaging software.

For immunofluorescence labelling of αSMA, sections were incubated with mouse‐on‐mouse blocking solution containing unconjugated AffiniPure Fab Fragment Donkey Anti‐Mouse IgG (Jackson ImmunoResearch, West Grove, PA, USA) in PBS for one hour at room temperature and then post‐fixed for 10 minutes in 4% PFA. Sections were incubated overnight at 4°C with the primary antibody αSMA diluted in PBS containing 0.1% BSA and 0.3% Triton X100. Hereafter, sections were washed for 30 minutes in PBS with 0.1% BSA, 0.2% gelatine and 0.05% saponin and then incubated with Alexa Flour 488‐conjugated secondary antibody (Life Technologies, Thermo Fisher Scientific, Waltham, MA, USA) at room temperature for 30 minutes. Then, counterstaining with 4,6‐diamindino‐2‐phenylindole (DAPI; diluted 1:200 in PBS, Thermo Fisher, D1306) was carried out and the sections were rinsed with PBS and mounted with SlowFade Gold Antifade Mountant (Life Technologies, Thermo Fisher Scientific, Waltham, MA, USA). Fluorescence microscopy was carried out using an Olympus BX61 microscope and the images were processed in Xcellence Rt software.

For immunofluorescent labelling of paraffin sections for EP_1_ and markers for TAL and CDs, labelling and imaging was carried our as previously described.^48^ In brief, 2 µm paraffin sections were deparaffinized overnight in xylene and subsequently rehydrated. Antigens were retrieved by boiling in TEG buffer (10 mM Tris pH 9, 0.5 mM EGTA) and aldehyde groups quenched by incubation in 50 mM NH4Cl in PBS for 30 minutes. Sections were blocked and permeabilized in 1% BSA, 0.2% gelatin and 0.05% saponin in PBS and labelled with rabbit‐anti EP_1_, followed by incubation with Alexa Fluor‐647‐conjugated antibody donkey‐anti‐rabbit and 2 μg/mL Hoechst33342. The sections were washed in PBS and mounted using glycergel mounting medium. For double stainings, goat‐anti‐AQP2 (sc‐9880 Santa Cruz Biotechnology) and sheep‐anti‐TAM (AB733 EMD Millipore Corp) were used with Alexa Fluor‐594‐conjugated secondary antibodies towards goat and sheep respectively. Control of secondary antibody specificity was performed. Imaging was performed on a Nikon Eclipse Ti‐E inverted microscope equipped with Plan Apo 60X (NA 1.40) oil objective, a Perfect Focus 3 system, a Zyla sCMOS5.5 Megapixel camera (Andor). The system was controlled by NIS Elements software from Nikon. The fluorescence illumination system was Cool LED‐pE‐300 white, and fluorescence filter sets were standard DAPI, GFP, TxRed and Cy5. Image analysis was performed using ImageJ Fiji software.^49^ Background and contrast were adjusted liniary.

Additionally, sections were stained with Mayers Hematoxylin for visualization of tubular dilation, and Picro Sirius Red and Masson's trichrome to visualize collagen deposition. The latter two stains were quantified in the mice study by capturing eight pictures from each slide at ×20 magnification and in the human slices by capturing five pictures from each slide at ×40 magnification. The fibrotic area was measured as percentage of total tissue area in ImageJ software. Light microscopy was carried out with the Olympus BX50 light microscope and the CellSens imaging software.

### 4.9 Statistics

Data were analysed in GraphPad Prism. Data with two or more parameters were analysed by two‐way ANOVA followed by Tukey's or Bonferroni's post‐hoc comparison. When data comprised of one parameter, One‐way ANOVA followed by Tukey's post hoc test or two‐tailed Students *t*‐test were used. Results were considered to be statistically significant when *P* < .05.

## CONFLICT OF INTEREST

The authors have declared that no conflicts of interest exist.

## AUTHOR CONTRIBUTIONS

J‐CK, HAMM, LNN, HP and RN designed the study. J‐CK, HAMM, MSJ, SJT, LNN, HP and RN carried out the experiments and analysed the data. MGM helped with human tissue procurement. J‐CK, HAMM, and RN wrote the manuscript with critical review from MSJ, SJT, MGM LNN and HP All of the authors approved the final version of the manuscript for publication.

## Supporting information

Fig S1

## Data Availability

The data that support the findings of this study are available from the corresponding author upon reasonable request.
