# Supplementary figures and images for: EP1 receptor antagonism mitigates early and late stage renal fibrosis
Source: Acta Physiol (Oxf). 2022 Jan 30;234(3):e13780. doi: 10.1111/apha.13780 (PMC9286353; doi:10.1111/apha.13780)

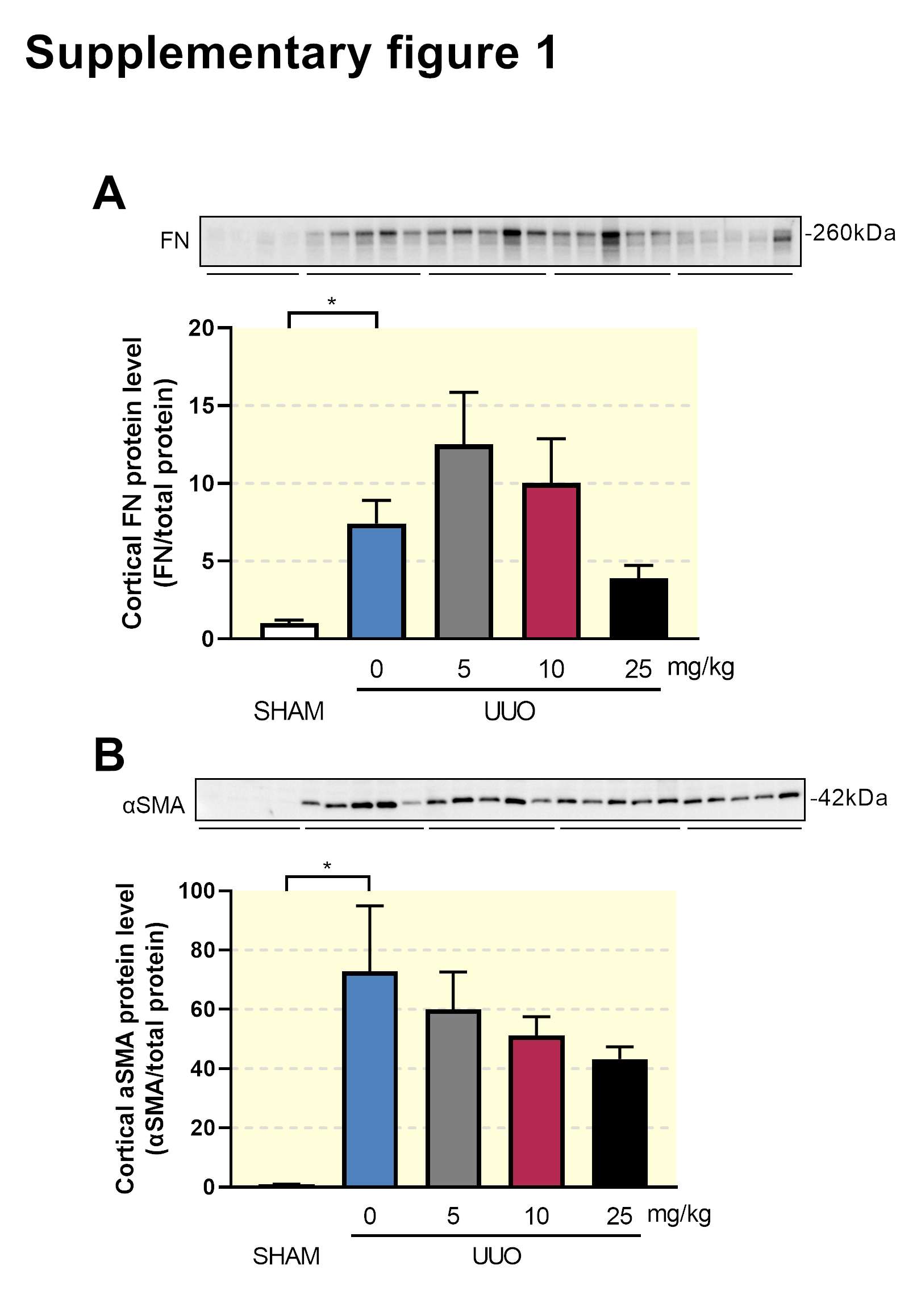

Supplement: Supplementary file 1 — Fig S1 [file APHA-234-0-s001.tif]
